# Association of Working Environment and Anxiety Levels in Clinical Professionals During the COVID-19 Pandemic

**DOI:** 10.7759/cureus.17450

**Published:** 2021-08-26

**Authors:** Aaiza Azhar, Amina Mahmood, Fahad Hasan, Ambreen Tauseef, Ayesha Shahzad, Taha Ahmed Tarin

**Affiliations:** 1 Internal Medicine, CMH Lahore Medical College and Institute of Dentistry, Lahore, PAK; 2 Medicine, CMH Lahore Medical College and Institute of Dentistry, Lahore, PAK; 3 Physiology, CMH Lahore Medical College and Institute of Dentistry, Lahore, PAK

**Keywords:** anxiety, clinical professionals, covid-19, zung anxiety scale, working environment

## Abstract

Introduction

Coronavirus disease 2019 (COVID-19) is a global pandemic and has become a major life-threatening challenge. The sudden and fast spread of the COVID-19 pandemic worldwide caused a sudden increase in the workload of health care workers in parallel with the possible increase in mortality rates and the spread of this disease to a large number of people. Clinicians, who are our frontline warriors, are not only at high risk of catching COVID-19, but their mental health is also at stake. The objective of this study was to determine the prevalence of anxiety and its association with the working environment in clinical professionals during the COVID-19 pandemic.

Methods

This cross-sectional study collected data from 400 medical doctors through an online survey, carried out for six months. The anxiety of participants was assessed by using the Zung Self Rating anxiety scale (SAS). An anxiety index of greater than or equal to 50 was marked as anxious. Descriptive chi-square analysis and correlation analysis were used.

Results

This study found that out of a total, 20.1% of the participants suffered from anxiety. Anxiety was found to be positively associated with sociodemographic factors like the age of the doctor (p=0.001), their gender (p=0.000), their working environment (p=0.005), working in basic healthcare units (p=0.015), patient load per week (p=0.005), personal protective equipment (PPE) availability to doctor according to WHO guidelines (p=0.007), and patient compliance with doctors’ orders (p=0.009).

Conclusion

We conclude that professional healthcare workers suffered from anxiety due to working conditions in the COVID-19 pandemic. Specific interventions and steps such as improving staffing and resources, policies to ensure fair distribution of working hours and rest breaks, workplace protections, work-family balance, health professional's emotional stability, and long-term benefits should be taken so as to minimize the lasting effects of these factors.

## Introduction

In December 2019, the first case of an unknown infection, which later came to be known as coronavirus disease 2019 (COVID-19), was reported in Wuhan, China, which caused an alarm not only on a national but also on an international scale. This was later declared by the World Health Organization (WHO) as a PHIEC (public health emergency of concern) [1].

The fast spread of the COVID19 pandemic has caused a massive increase in the workload of health care workers. Clinicians, who are our frontline warriors, are not only at high risk of catching COVID-19, but their mental health is also at stake. Due to a lack of knowledge surrounding the severity and extent of this issue, there can be serious detrimental effects on the morale and motivation of that healthcare worker dealing directly with this issue. Studies have revealed high rates of anxiety and distress symptoms in clinical professionals during the ongoing pandemic [2].

In Pakistan, the first case of COVID-19 was reported on February 26, 2020, and since then, the death toll has been staggering [3]. Pakistan being a developing country, already has a fragile healthcare system, with a workforce not enough to cater to the needs of the huge number of patients, poor hospital infrastructure, and lack of basic equipment and facilities [4]. The COVID-19 pandemic has just added to the already long list of problems within Pakistan’s healthcare system. The initial lack of testing equipment/kits also contributed to the unawareness of the scale of this pandemic and led to the rapid spread of the disease [5].

Prolonged exposure to large numbers of patients (potentially infected or confirmed cases), lack of personal protective equipment, and poor patient compliance to orders such as “wear a mask” increase the risk of infection for the health professional community, which may enhance their anxiety and stress levels. Furthermore, physicians have to isolate themselves from family and friends in order to prevent them from being infected, and this also has the potential to increase their anxiety levels.

Previously, a few studies were conducted to assess the psychical impact of this on health care workers in Pakistan [6], however, they either dealt with negative psychological impact in general and regarding anxiety, its prevalence, and its association with a few “demographic” variables (not working conditions) was studied. Furthermore, none of them looked specifically into patient compliance to simple orders like “wear a mask” or “bring one attendant only,” and its effect on the anxiety of clinicians. Similarly, doctors isolating themselves after coming back from work has not been studied as a potential for causing anxiety [7].

Similarly, health workers play an important role in combating the pandemic, especially in third-world countries with poorly managed health systems. They are social heroes and having borne the brunt of this pandemic, whereas most are also exposed largely to devastating effects. This study will help to find the association of current working conditions in health care centers contributing to/affecting the mental health/anxiety levels of doctors so that, in the future, we can better plan to provide facilities to aid workers.

## Materials and methods

After getting approval from the institutional ethical review board, data were collected by 400 participants via online questionnaires in 2020. Online consent was taken, and eligibility criteria were checked before data entry. All the clinical health professionals/consultants (doctors only) of either gender, residing in major cities of Punjab, were included. All those subjects who were already using any medication for depression, anxiety, or any other psychological disorder were excluded.

After taking their consent, the second part recorded their sociodemographic information (age, gender, etc.) while the third part dealt with their professional details (year’s active, specialty, etc.). The next few questions are regarding their working conditions (working hours, patient load, personal protective equipment (PPE) availability, etc.), and medical status (comorbidities). Lastly, a series of questions were added to assess their anxiety levels from the Zung self-rating anxiety scale (SAS). The cut-off value was set at 50, whereas an anxiety index below 50 was considered normal and above 50 was considered anxious.

Statistical analysis

To test the objectives of the study, data were statistically analyzed using SPSS software version 25 (IBM Corp., Armonk, NY). Data were analyzed for normality by using the Shapiro-Wilk test. Median with IQR was used for non-symmetrical data, whereas mean ± SD was used for symmetrical data. The relationship of all variables with anxiety levels was correlated. Descriptive statistical methods, chi-square analysis, and correlation analysis were used. The analysis was carried out at a confidence range of 95%, and a p-value ≤ of 0.05 was considered statistically significant.

## Results

Out of 400 surveys, we found a positive association between anxiety and age (p = 0.001), especially in the age group less than 25 (37.5%), and gender (p= 0.000) where female doctors (28%) were found to be more anxious than male doctors (11.8%). A statistically significant association was also found between anxiety and the social background of the doctor (p=0.054, Table 1).

**Table 1:** Anxiety according to age groups and sociodemographic status of health professionals in the working environment *p-value ≤0.05 statistically significant

Variable		Anxiety present n(%)	Anxiety absent n(%)	Total frequency n(%)	p-value
Age	<25	12 (37.5)	20 (62.5)	32 (10.2)	*0.001
	25-30	26 ( 24.5)	80 (75.5)	106 (33.9)	
	31-40	17 (27.4)	45 (72.6)	62 (19.8)	
	41-50	4 (8)	46 (92)	50 (16)	
	51-60	3 (6.1)	46 (93.9)	49 (15.7)	
	>60	1 (7.1)	13 (92.9)	14 (4.5)	
Gender	Male	18 (11.8)	134 (88.2)	152 (48.6)	*0.000
	Female	45(28)	116 (72)	161 ( 51.4)	
Social background	Rural	3 (8.3)	33 (91.7)	36 (11.5)	*0.054
	Urban	47 (20.1)	187 (80)	234 (74.8)	
	Semi-urban	13 (30.2)	30 (69.8%)	43 (13.7)	
Marital status	Single	24 (23.1)	80 (76.9)	104 (33.2)	0.359
	Married	39 (18.7)	170 (81.3)	209 (66.8)	

It was observed that the more years these health professionals remained active in the medical profession, the more they suffered from anxiety (p=0.005). Moreover, those doctors who worked in basic health units (p=0.015) and had a bigger patient load (>250 patients/week) suffered more from anxiety (p=0.005).

Those clinical professionals who were not provided with proper PPE were more anxious during their duty timings (p=0.007). In addition, patient compliance with doctors’ orders was also one of the factors linked to the development of anxiety (p=0.009). The medical status of a doctor (having chronic conditions like stroke, hypertension, diabetes, etc) was found to have no significant association with the development of anxiety, except for migraine (p=0.000). Likewise, this study showed no statistically significant association of prolonged duty hours/week (p=0.727), specialty (p=0.257), duty appointment in COVID-19 diagnostic camps (p=0.571), and limitation with family interactions (p=0.600) with anxiety (Table 2).

**Table 2:** Anxiety according to different factors in the working environment *p-value ≤0.05 statistically significant

Variable			Anxiety present n(%)	Anxiety absent n(%)		Total frequency n(%)	p-value
Specialty	Anesthesia and ICU		2 (18.2)	9 (81.8)		11 (3.5)	0.257
	Pathology		12 (27.9)	31 (72.1%)		43 (13.7)	
	Internal medicine		9 (13.8)	56 (86.2)		65 (20.8)	
	Community medicine		0 (0.0)	5 (100)		5 (1.6)	
	Surgery		2 (10)	18 ( 90)		20 (6.4)	
	General physician		8 (17.4)	38 (82.6)		46 (14.7)	
	Gynae/obs		1 (7.7)	12 (92.3)		13 (4.2)	
	Pediatrics		6 (35.3)	11 (64.7%)		17 (5.4%)	
	Radiology		2 ( 22.2)	7 (77.8)		9 (2.9)	
	Emergency medicine		2 (33.3)	4 (66.7)		6 (1.9)	
	Dermatology		0 (0.0)	6 (100%)		6 (1.9%)	
	Ophthalmology		0 (0.0)	6 (100)		6 (1.9)	
	Urology		0 (0.0)	1 (100)		1 (0.3)	
	Dentistry		4 (28.6)	10 (71.4)		14 (4.5)	
	None ( house job )		15 (29.4)	36 ( 70.6)		51 (16.3)	
Years active in medical service	<2 years		13 (26)	37 (74)		50 (16)	*0.005
	2-5 years		23 (30.7)	52 (69.3)		75 (24)	
	6-10 years		10 (23.3)	33 (76.7)		43 (13.7)	
	>10 years		17 ( 11.7)	128 (88.3)		145 (46.3)	
Setup (works in basic healthcare unit BHU )	Yes		10 ( 38.5)	16 (61.5)		26 (8.3)	*0.015
	No		53 ( 18.5)	234 (81.5)		287 (91.6)	
Hours at duty per week	< / = 35 hours		21 (20.6)	81 (79.4)		102 (32.6)	0.727
	36-95 hours		36 (19.5)	149 (80.5)		185 (59)	
	96-125 hours		4 (19.0)	17 ( 81)		21 (6.7)	
	126-175 hours		2 (40)	3 (60)		5 (1.6)	
Number of patients seen per week	<25		8 (14.5)		47 (85.5)	55 (17.6)	*0.005
	25-50		10 (16.4)		51(83.6)	61 (19.5)	
	51-100		9 (14.1)		55(85.9%)	64 (20.4%)	
	101-150		7 (24.1)		22(75.9)	29 (9.3)	
	151-200		2 (7.7)		24(92.3)	26 (8.3)	
	201-250		3 (30.0)		7(70.0)	10 (3.2)	
	250 +		12 (48)		13(52.0)	25 (8.0)	
	Not applicable ( no contact )		12(27.9)		31(72.1)	43 (13.7)	
Workload	Increased		11 (15.9)		58 (84.1)	69 (22)	0.119
	Decreased		31 (18.2)		139 (81.8)	170 (54.3)	
	Same as before		21(28.4)		53 (71.6)	74 (23.6)	
Availability of PPE	Everyday		18 (13.5)		115 (86.5)	133 (42.5)	*0.007
	Most of the days		16 (18.4)		71 (81.6)	87 (27.8)	
	Rarely		19 (28.4)		48 (71.6)	67 (21.4)	
	never		10 (38.5)		16 (61.5)	26 (8.3)	
Is PPE availability according to WHO guidelines?	Yes		26 (14.4)		154 (85.6)	180 (57.5)	*0.008
	No		25 (25.5)		73 (74.5)	98 (31.3)	
	Not applicable		12 ( 34.3)		23 (65.7)	35 (11.2)	
Patient compliance	Never		5 (23.8)		16 (76.2)	21 (6.7)	*0.009
	Seldom		21 (34.4)		40 (65.6)	61 (19.5)	
	Sometimes		9 (17.6)		42 (82.4)	51 (16.3)	
	Often		16 (16.0)		84 (84)	100 (31.9)	
	Always		5 (8.8)		52 (91.2)	57 (18.2)	
	Not applicable		7 (30.4)		16 (69.6)	23 ( 7.3)	
Limitation to interaction with family members	Yes		52 (22.7)		177 (77.3)	229 (73.2)	0.060
	No		11 ( 13.1)		73 (86.9)	84 (26.8)	
Medical status ( person has migraines )	Yes		13 (48.1)		14 (51.9)	27 (8.6)	*0.000
	No		50 (17.5)		236 (82.5)	286 (91.3)	

## Discussion

This study found a positive association between the working environment and anxiety levels in clinical professionals during the COVID-19 pandemic in Pakistan. We used a cut-off anxiety index of 50 (40 on raw score) as used by a recent study conducted by Dunstan and Scott [8] instead of using a cut-off score of 36 as previously employed. This is also useful in preventing false-positive results while using the Zung self-rating anxiety scale (SAS).

This study found a high prevalence of anxiety in females as compared to their male colleagues. This is consistent with a study conducted by Atif Khan [9], which had also found that female doctors to be generally more prone to develop anxiety and depression, and psychological stress. This might be due to high expectations, multiple roles, and a work environment. Differences in physiological and psychological aspects of coping with stress have also been linked to these differences [10].

In Pakistan, the healthcare system has been divided into the government sector, private sector, and basic health units (BHU). Out of all, only participants in our study working primarily in BHU showed a link with their setup and anxiety, whereas no such link was seen in doctors in others setups specified above. This could be possibly credited to different factors such as lack of availability of sufficient staff and necessary medical equipment, factors that in a crisis such as the current pandemic are of vital importance for the functionality of doctors.

Physicians younger than 35 years of age were more likely anxious than senior doctors. This was also found by Amin Sharif [11] who while, assessing the link of the same factors with COVID-19 reported a similar result. Doctors with work hours ranging between 36 and 95 hours per week and having a patient load of around 51-100 per week were at a high frequency, and there was indicated to be a significant statistical link of patient load per week with the development of anxiety. This could insinuate a link that long hours in an environment where there is a high chance of exposure to a large number of patients who might possibly be infected may create a sense of distress that could ultimately lead to lasting effects on the physical and psychological health of doctors. Our data reported a high percentage of anxious doctors to have a patient load exceeding 250 patients per week. Working hours were not found to be a risk factor for anxiety in analysis, opposite to findings of a study that linked working hours greater than 60 hours per week as a risk factor for developing depressive and anxiety symptoms [12].

The current study did not find any statistically significant association between workload and its effect on anxiety; however, in previous literature, an increased workload has been identified as providing way as an easy target of exhaustion and cumulative to depression and anxiety as found by Chen Liu [13].

Frontline doctors working in COVID-19 camps and having their majority work consisting of dealing with high-risk patients are found to be more prone to depressive and anxious episodes than their non-frontline working colleagues as indicated by a survey conducted in China during the coronavirus pandemic [14]. A considerable majority of our participants were not employed in COVID camps and hence no association was found with anxiety.

To the best of our knowledge, this factor and behavior, which indirectly affect the psychological health of doctors dealing with these patients, is something that has not yet been studied so far, but one could presume that negative response on part of the patient could add to the stress of doctor dealing with them. This, along with the above factors, can be a significant driving force to mental health issues.

An unusual yet accounted for fact was the fear of spreading the infection to family members by those actively in contact with COVID-19-positive patients and even in those having no active contact with these patients in a hospital environment. This created the need for these healthcare workers to isolate themselves from their family and have minimal physical interaction with them, which negatively impacted and disrupted their mental well-being, creating a sense of loneliness in times when familial support is of utmost importance. A study conducted by Urooj Ansari [15] showed 79.7% of its participants (doctors) faced this same fear, similar to what our analysis reported (73%).

All the above-stated factors corroborate with a study carried out by Shanafelt Ripp [16], pointing out the sources of anxiety among healthcare workers after increased intensity and duration of work increases the chances of early burnout.

Limitations

Similar, large-scale studies are needed to better address the mental health impact of the COVID-19 pandemic.

## Conclusions

We found a prevalence and positive association between professional healthcare workers and anxiety during working conditions in the COVID-19 pandemic. This emphasizes providing a safe working environment to frontline healthcare workers in order to ensure their mental well-being.
